# Preexisting Dementia Is Associated with Increased Risks of Mortality and Morbidity Following Major Surgery: A Nationwide Propensity Score Matching Study

**DOI:** 10.3390/ijerph17228431

**Published:** 2020-11-14

**Authors:** Yu-Ming Wu, Hsien-Cheng Kuo, Chun-Cheng Li, Hsiang-Ling Wu, Jui-Tai Chen, Yih-Giun Cherng, Tzeng-Ji Chen, Ying-Xiu Dai, Hsin-Yi Liu, Ying-Hsuan Tai

**Affiliations:** 1 Department of Anesthesiology, Shuang Ho Hospital, Taipei Medical University, New Taipei City 23561, Taiwan; b101096087@yahoo.com.tw (Y.-M.W.); alwayswhite0224@gmail.com (H.-C.K.); hamelmorris@gmail.com (C.-C.L.); 19240@s.tmu.edu.tw (J.-T.C.); stainless@s.tmu.edu.tw (Y.-G.C.); cindyliu5652@gmail.com (H.-Y.L.); 2 Department of Anesthesiology, School of Medicine, College of Medicine, Taipei Medical University, Taipei 11031, Taiwan; 3 Department of Anesthesiology, Taipei Veterans General Hospital, Taipei 11217, Taiwan; xianling30@gmail.com; 4 School of Medicine, National Yang-Ming University, Taipei 11217, Taiwan; tjchen@vghtpe.gov.tw (T.-J.C.); daiinxiu@gmail.com (Y.-X.D.); 5 Department of Family Medicine, Taipei Veterans General Hospital, Taipei 11217, Taiwan; 6 Department of Dermatology, Taipei Veterans General Hospital, Taipei 11217, Taiwan

**Keywords:** Alzheimer’s disease, complication, infection, outcome, vascular dementia

## Abstract

Patients with dementia are predisposed to multiple physiological abnormalities. It is uncertain if dementia associates with higher rates of perioperative mortality and morbidity. We used reimbursement claims data of Taiwan’s National Health Insurance and conducted propensity score matching analyses to evaluate the risk of mortality and major complications in patients with or without dementia undergoing major surgery between 2004 and 2013. We applied multivariable logistic regressions to calculate adjusted odds ratios (aORs) with 95% confidence intervals (CIs) for the outcome of interest. After matching to demographic and clinical covariates, 7863 matched pairs were selected for analysis. Dementia was significantly associated with greater risks of 30-day in-hospital mortality (aOR: 1.71, 95% CI: 1.09–2.70), pneumonia (aOR: 1.48, 95% CI: 1.16–1.88), urinary tract infection (aOR: 1.59, 95% CI: 1.30–1.96), and sepsis (OR: 1.77, 95% CI: 1.34–2.34) compared to non-dementia controls. The mortality risk in dementia patients was attenuated but persisted over time, 180 days (aOR: 1.49, 95% CI: 1.23–1.81) and 365 days (aOR: 1.52, 95% CI: 1.30–1.78) after surgery. Additionally, patients with dementia were more likely to receive blood transfusion (aOR: 1.32, 95% CI: 1.11–1.58) and to need intensive care (aOR: 1.40, 95% CI: 1.12–1.76) compared to non-dementia controls. Senile dementia and Alzheimer’s disease were independently associated with higher rates of perioperative mortality and complications, but vascular dementia was not affected. We found that preexisting dementia was associated with mortality and morbidity after major surgery.

## 1. Introduction

The latest global estimate shows that dementia is a leading global epidemic, affecting approximately 47 million individuals worldwide [1]. Dementia leads to dependence, impaired quality of life, institutionalization, and mortality, which causes a growing and substantial burden of disease in the aging population [1]. Epidemiologic study has reported that the number of people affected by dementia is expected to increase to 131 million by 2050 [2]. Given this demographic trend, we can expect a growing number of dementia patients undergoing surgery in the future. Dementia patients undergoing surgery experience readmission more commonly and generally have longer lengths of hospital stay compared to those without dementia [3], which poses a serious challenge to the healthcare system in perioperative care. 

In addition to memory loss and cognitive decline, patients with advanced dementia are predisposed to specific physiological abnormalities, including dysphagia, weight loss, wasting syndrome, susceptibility to infection, and sensory abnormality [4,5]. These pathologies may potentiate a higher perioperative risk of mortality and morbidity in the setting of major surgery. However, whether dementia associates with higher rates of perioperative mortality and morbidity remains uncertain due to several study limitations in previous reports, including small sample size [6,7,8,9], mixed groups of different dementia subtypes [6,7,8,9,10,11], inadequate consideration for confounding factors [8], disadvantages of unmatched statistical methodology [6,7,8,9,10,11], and restriction to specific types of surgery [7,8,10,11] or hospitals [6,7,8,9]. Clarifying the impact of dementia on perioperative risk is of great value to risk stratification and optimal care for this susceptible population.

Accordingly, we conducted a nationwide population-based cohort study to evaluate the risk of mortality and morbidity in patients with dementia undergoing major surgery. Based on the evidence of prior studies [6,7,8,9,10,11], we hypothesized that preexisting dementia is associated with greater risks of all-cause in-hospital mortality and complications after major surgery.

## 2. Materials and Methods

### 2.1. Source of Data

We obtained approval from the Institutional Review Boards of Taipei Veterans General Hospital and Taipei Medical University in Taiwan (TMU-JIRB-N202010042 and IRB-TPEVGH-2013-04-005E). Written informed consent was waived by the Institutional Review Board. The Taiwan National Health Insurance program was established in March 1995 and covered more than 99% of 23.4 million Taiwanese residents at the end of 2013. The National Health Insurance (NHI) research database contains comprehensive data of the insured individuals, including demographic characteristics (date of birth, sex, and residential location) and claims data (outpatient and inpatient care, physicians’ primary and secondary diagnoses, prescriptions, treatment procedures, and medical expenditures) [12,13,14,15,16,17]. 

### 2.2. Study Design

We utilized three Longitudinal Health Insurance Databases (LHID2000, LHID2005, and LHID2010) which randomly sampled 1 million beneficiaries from the original NHI research database in the years 2000, 2005, and 2010, respectively. The LHIDs contain the most updated medical claims of sampled subjects since 1997. The representativeness of LHIDs has been validated by Taiwan’s National Health Research Institutes [18]. We selected individuals aged more than 20 years who underwent a first major surgery requiring hospitalization for ≥2 days between January 2004 and November 2013 to identify patients with a history of dementia within 24 months prior to the index surgery. Each patient with dementia was randomly matched to a surgical patient without a history of dementia, using a frequency matched pair procedure (case–control ratio, 1:1) to adjust for age, sex, income, coexisting diseases, types of surgery, types of anesthesia, and number of hospitalizations and emergency visits within 24 months prior to the index surgery. We compared the risk of postoperative mortality and morbidity after the index surgery between patients with and without preexisting dementia. 

### 2.3. Ascertainment of Dementia

We identified patients with dementia using the International Classification of Diseases, 9th Revision, Clinical Modification (ICD-9-CM) codes. This includes senile dementia (ICD-9-CM 290.0-290.3), vascular dementia (ICD-9-CM 290.4), Alzheimer’s disease (ICD-9-CM 331.0), and other subtypes of dementia (ICD-9-CM 294.1) [17]. The diagnoses of dementia were made at least 2 times by board-certified neurologists or psychiatrists. We also evaluated the medications for dementia (i.e., cholinesterase inhibitors or *N*-methyl-d-aspartate (NMDA) receptor antagonists) within 24 months prior to the index surgery, including donepezil, galantamine, rivastigmine, and memantine [19]. In Taiwan’s NHI regulations, treatment for dementia is reimbursable only after comprehensive laboratory and imaging studies to exclude neurocognitive impairments from other etiologies, including vitamin B12 deficiency, thyroid disease, or cerebrovascular disease.

### 2.4. Covariate and Outcome Measurement

We used the ICD-9-CM codes of physicians’ diagnoses within 24 months prior to surgery to identify the history of the following coexisting diseases, chosen based on data availability, physiological plausibility, and the existing literature: hypertension, diabetes mellitus, ischemic heart disease, atherosclerosis, cardiac dysrhythmias, heart failure, liver cirrhosis, chronic obstructive pulmonary diseases, chronic kidney disease, cerebrovascular disease, Parkinson’s disease, malignancies, sarcopenia, and obesity. (Supplementary Material) Monthly premium was classified into $0–$500, $501–$800, and >$800 United States dollars (USD).

The primary outcome was 30-day all-cause in-hospital mortality, which was chosen based on a wide range of historical surgical disciplines [20] and data availability [18]. However, to measure long-term outcomes of dementia patients following surgery, we also analyzed 180-day and 365-day all-cause in-hospital mortality in this study. Secondary outcomes were determined from medical claims that occurred within 30 days after index admission, including pneumonia, urinary tract infection, pyelonephritis, surgical site infection, sepsis, acute myocardial infarction, stroke, pulmonary embolism, deep vein thrombosis, cardiac dysrhythmias, acute renal failure, and postoperative bleeding. The NHI research database has been widely validated to evaluate the validity of diagnosis codes and to develop study methodologies, including pneumonia [21], acute myocardial infarction [22], and ischemic stroke [23]. We also collected data about the requirements for blood transfusion [24,25,26] and intensive care during the index surgical admission. 

### 2.5. Statistical Analysis

We conducted matching procedures with propensity score to balance the distribution of age, sex, monthly premium, coexisting diseases, and numbers of emergency visits and hospitalizations within 24 months before surgery between surgical patients with and without dementia. A non-parsimonious multivariable logistic regression model was applied to estimate a propensity score for subjects with or without dementia. We matched dementia subjects to non-dementia controls using a greedy matching algorithm without replacement and within a tolerance limit of 0.05 [27]. Categorical variables were summarized using frequency (percentage), and continuous variables were summarized using mean ± standard deviation or median (interquartile range). The distributions of baseline characteristics in propensity-score-matched samples were compared between subjects with and without dementia by using standardized difference [28]. In this study, we defined perioperative risk as the rate, proportion, or probability of mortality and complications listed in Section 2.4 for dementia patients and controls. Adjusted odds ratios (aORs) and 95% confidence intervals (CIs) of postoperative mortality and complications were calculated by multivariable logistic regressions. Sensitivity analyses were performed to avoid possible collapsibility bias and multicollinearity from the propensity score matching algorithm, including exclusions of subjects who had dementia diagnosis <1 year before surgery, who underwent cardiovascular surgery, or who were diagnosed with any type of cancer before surgery. We also conducted stratified analyses by age, sex, subtypes of dementia, uses of dementia medication, duration of dementia diagnosis before surgery, and types of anesthesia to examine perioperative risk among patients with dementia within these strata. We considered a two-sided level of 0.05 statistically significant. All statistical analyses were conducted using Statistics Analysis System (SAS), version 9.4 (SAS Institute Inc., Cary, NC, USA).

## 3. Results

**Figure 1:**
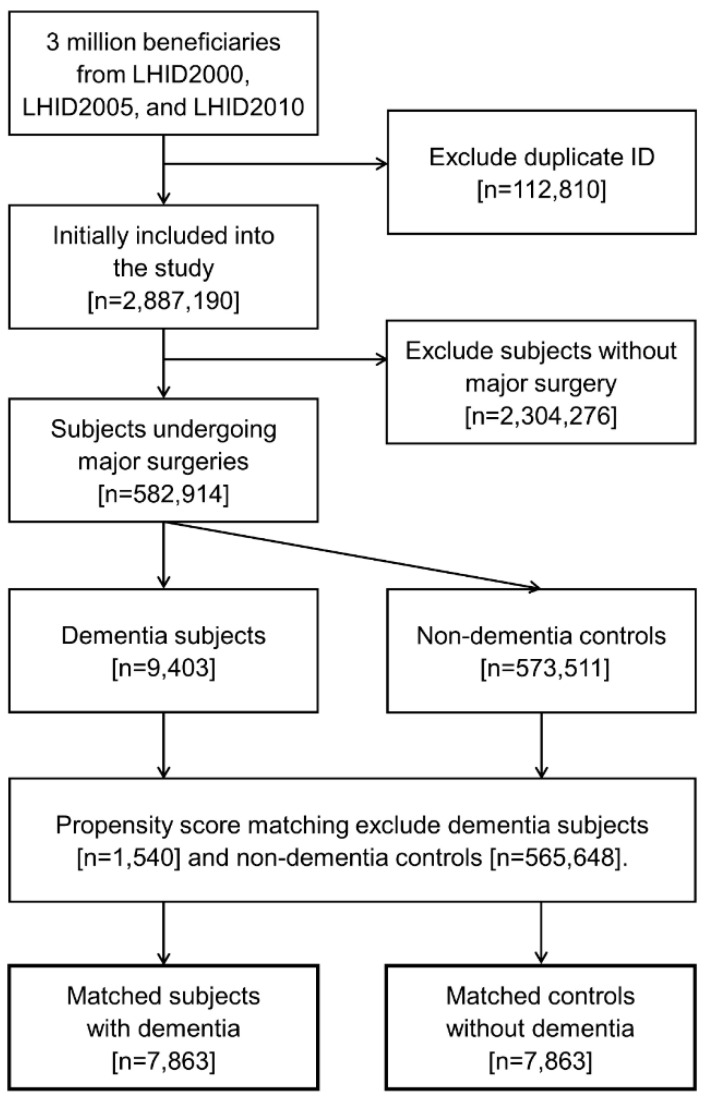
Flow diagram for patient selection.

After the matching process, a total of 7863 matched pairs were included for multivariable logistic analyses (Figure 1). Table 1 shows the baseline characteristics of the included subjects with and without dementia. The distributions of demographics, coexisting diseases, types of surgery, and anesthesia were well balanced after propensity score matching. The duration of dementia diagnosis before surgery was median 2.3 years (interquartile range 1.0–4.3 years). Among the dementia subjects, 696 (8.9%) of them received reimbursable medications for dementia and 55 (0.7%) and 4 (0.1%) used two and three types of dementia medications within 24 months before surgery, respectively. 

### 3.1. Risk of Postoperative Mortality and Morbidity

There were 52 (0.7%) and 30 (0.4%) in-hospital deaths that occurred within 30 days after surgery in dementia subjects and non-dementia controls, respectively. After adjusting for covariates, preexisting dementia was significantly associated with 30-day in-hospital mortality (aOR: 1.71, 95% CI: 1.09–2.70) compared to non-dementia controls. (Table 2) Sensitivity analyses demonstrated similar results. (Table 3) Dementia was also associated with higher risks of 180-day (aOR: 1.49, 95% CI: 1.23–1.81) and 365-day all-cause in-hospital mortality (aOR: 1.52, 95% CI: 1.30–1.78) after surgery. 

Dementia was also significantly associated with greater risks of pneumonia (aOR: 1.48, 95% CI: 1.16–1.88), urinary tract infection (aOR: 1.59, 95% CI: 1.30–1.96), and sepsis (aOR: 1.77, 95% CI: 1.34–2.34) compared to non-dementia controls. Additionally, patients with dementia were more likely to receive blood transfusion (aOR: 1.32, 95% CI: 1.11–1.58) and to need intensive care (aOR: 1.40, 95% CI: 1.12–1.76). (Table 2).

### 3.2. Stratified Analyses by Age, Sex, and Subtypes of Dementia

Table 4 shows the results of perioperative risk among patients with dementia, stratified by age, sex, subtypes of dementia, uses of dementia medications, duration of dementia diagnosis, and types of anesthesia. The perioperative risk associated with dementia was significant in subjects with age ≥65 years (aOR: 1.26, 95% CI: 1.13–1.40) and female (aOR: 1.35, 95% CI: 1.16–1.56). As for dementia subtype, senile dementia and Alzheimer’s dementia were both associated with higher perioperative risk, aOR: 1.27 (95% CI: 1.14–1.42) and aOR: 1.45 (95% CI: 1.07–1.96), respectively. The perioperative risk was consistently increased in dementia patients receiving general anesthesia (aOR: 1.28, 95% CI: 1.11–1.47) and regional anesthesia (aOR: 1.32, 95% CI: 1.09–1.60).

## 4. Discussion

This study discovered that dementia is independently associated with higher risks of 30-day in-hospital mortality, pneumonia, urinary tract infection, and sepsis following major surgery compared to non-dementia controls. In addition, patients with dementia were prone to blood transfusion and admission to intensive care during their surgical hospitalizations. As for subtype of dementia, we found that senile dementia and Alzheimer’s disease but not vascular dementia are linked to mortality and morbidity following major surgery. 

Our results were based on a nationwide dataset and covered various types of surgery. In contrast, most prior studies restricted the study population to single hospital [6,7,8,9] and orthopedic [7,10,11] or vascular surgery [8], which made it difficult to generalize their results to institutions with different settings or other types of surgery. Our analyses have taken the subtypes and medications of dementia into account and implemented propensity score matching to best control for potential confounding effects, which was lacking in previous studies [6,7,8,9,10,11]. We observed a significantly higher risk of 30-day all-cause in-hospital mortality, in line with prior studies [6,7,8,9,10]. The increased risk of mortality associated with dementia was attenuated over time but persisted for 180 and 365 days after surgery, which has not been reported previously [6,7,8,9,10,11]. This finding suggests that preexisting dementia adversely impacts both short-term and long-term recovery after major surgery and that its disease burden is not merely limited to postoperative critical care but also to extended care facilities [10,29]. In addition, we also found that the rate of infection (e.g., pneumonia, urinary tract infection, and sepsis) but not thromboembolism (e.g. myocardial infarction and stroke) was significantly increased in dementia patients, in agreement with previous studies [9,11]. Of note, our study first demonstrated a higher likelihood of blood transfusion and intensive care in dementia patients. Studies have shown that dementia is a risk factor for development of perioperative delirium and vice versa [9,30]. In addition to dementia, unrecognized mild cognitive impairment was common in elderly people awaiting surgery [31]. The insult of major surgery may accelerate the progression of cognitive decline in this population [32]. Occurrence of perioperative delirium is an established predictor for both physical and cognitive morbidity after surgery [30,33]. Furthermore, delirium is an exceedingly costly complication, both to patients and to medical facilities. The occurrence of postoperative delirium significantly increases healthcare costs by an estimated 3000 to 10,000 USD additional cost per case [34,35]. The relationship between anesthesia and delirium is not yet fully elucidated. It remains controversial if anesthetic technique and monitoring are correlated with perioperative delirium [36,37,38,39,40]. Some studies claimed that the light depth of sedation and regional anesthesia are effective in preventing postoperative delirium [36,37]. However, a recent randomized trial showed that electroencephalogram-guided anesthetic administration did not reduce the incidence of postoperative delirium among elderly patients [38]. Besides, there is no adequate evidence supporting that the type of anesthesia affects postoperative delirium [39]. Regarding postoperative sedation, Djaiani and colleagues demonstrated that dexmedetomidine sedation decreased incidence and duration of delirium in older patients undergoing cardiac surgery compared to propofol [40]. Future studies are warranted to develop strategies to prevent perioperative delirium and its adverse effect on dementia patients.

We raised three possible explanations for the greater mortality and morbidity associated with dementia. First, dementia is closely linked to immune dysfunction and systemic inflammation, especially in Alzheimer’s disease [41,42]. Patients with advanced dementia are susceptible to infection and have a higher risk of infection-associated death [4,5,43]. Second, Alzheimer’s disease is a strong risk factor for anemia and vice versa [44,45]. This may predispose dementia patients to perioperative use of blood transfusion. Third, dementia is associated with impaired lung function and lung disease [46], which may increase the risk of respiratory complications and the need for intensive care following major surgery.

Importantly, our stratified analyses indicated that dementia of vascular origin does not produce an additional risk of postoperative mortality and morbidity compared to non-dementia counterparts. People with vascular dementia decline in their functional abilities at a slower rate compared to those with Alzheimer’s disease [47]. The better general health condition of subjects with vascular dementia might decrease the perioperative risk. Additionally, a study has reported that pneumonia-caused death was higher in Alzheimer’s disease compared to vascular dementia [48], suggesting a potential difference in patients’ respiratory and immune functions among distinct subtypes of dementia. The development of vascular dementia is closely associated with atherosclerosis and cardiovascular diseases (e.g., hypertension and cerebrovascular disease) [49,50], and these vascular risk factors have been largely controlled in our statistical analyses. Our findings highlight that the subtype of dementia should be taken into consideration in risk stratification for dementia patients undergoing surgery.

There are limitations to our study. First, our data did not contain information about physical measures, biochemical laboratory measures, detailed surgical and anesthetic procedures, perioperative medications, and physicians’ personal skills that were not covered by the NHI database. Therefore, we could not further adjust for cardiac and pulmonary functions; perioperative uses of antibiotics, immunosuppressants, and anti-thrombin therapy; serum hemoglobin level; and surgical blood loss, which might affect the occurrence of mortality, infection, and thromboembolism and the need for blood transfusions after surgery. Second, functional ability and nutritional status of included subjects were unknown in our study, which might also influence the survival of dementia patients [51,52]. Third, we could not differentiate between early and advanced dementia. Fourth, we could not evaluate the relationship between dementia and postoperative delirium due to data unavailability. Finally, residual confound is possible although our analyses have adjusted for a variety of potential confounding factors.

## 5. Conclusions 

We found that preexisting dementia was independently associated with mortality and morbidity after major surgery compared to non-dementia controls. The higher risk of mortality in dementia patients persisted for at least one year after surgery. Senile dementia and Alzheimer’s disease were associated with higher perioperative risk, but vascular dementia was not affected. Patients with dementia clearly require careful preoperative screening and tailored postoperative interventions to improve recovery after surgery. Our results highlight the need to further develop strategies of risk stratification and optimal perioperative care for this susceptible population.

## Figures and Tables

**Table 1:** Baseline characteristics of dementia cases and matched controls.

Baseline Characteristic	Dementia *n* = 7863		Control *n* = 7863		Standardized Difference ^†^
Age (years), mean (SD)	77.0	10.0	77.4	10.0	−0.0400
Sex, n (%)					0.0141
Male	3741	47.6	3691	46.9	
Female	4122	52.4	4172	53.1	
Monthly premium (USD), n (%)					0.0143
0–500	5684	72.3	5698	72.5	
501–800	2002	25.5	2030	25.8	
≥801	177	2.3	135	1.7	
Medication for dementia, n (%)					
Donepezil	375	4.8	0	0.0	NA
Galantamine	56	0.7	0	0.0	NA
Rivastigmine	276	3.5	0	0.0	NA
Memantine	52	0.7	0	0.0	NA
Comorbidity, n (%)					
Hypertension	5349	68.0	5388	68.5	−0.0126
Diabetes	2702	34.4	2732	34.7	−0.0093
Ischemic heart disease	2320	29.5	2293	29.2	0.0091
Atherosclerosis	249	3.2	262	3.3	−0.0290
Cardiac dysrhythmias	1210	15.4	1202	15.3	0.0043
Heart failure	1059	13.5	1041	13.2	0.0109
Liver cirrhosis	196	2.5	192	2.4	0.0117
COPD	1988	25.3	1921	24.4	0.0252
Chronic kidney disease	710	9.0	647	8.2	0.0561
Cerebrovascular disease	3466	44.1	3489	44.4	−0.0065
Parkinson’s disease	910	11.6	714	9.1	0.1491
Malignancy	914	11.6	876	11.1	0.0264
Sarcopenia	74	0.9	73	0.9	0.0076
Obesity	24	0.3	21	0.3	0.0738
Type of anesthesia, n (%)					0.0098
General anesthesia	4289	54.5	4298	54.7	
Regional anesthesia	2320	29.5	2359	30.0	
Other	1254	16.0	1206	15.3	
Type of surgery, n (%)					0.0132
Orthopedic	2845	36.2	2875	36.6	
Cardiovascular	892	11.3	864	11.0	
Neurosurgery	908	11.5	935	11.9	
Gastrointestinal	651	8.3	661	8.4	
Hepato-biliary-pancreatic	376	4.8	365	4.6	
Genitourinary	848	10.8	867	11.0	
ENT	287	3.7	287	3.7	
Gynecology	147	1.9	145	1.8	
Breast	57	0.7	53	0.7	
Other	852	10.9	811	10.4	
Number of hospitalizations, n (%)					0.1142
0	3953	50.3	4364	55.5	
1	1832	23.3	1755	22.3	
≥2	2078	26.5	1744	22.2	
Number of ER visits, n (%)					0.1311
0	2706	34.4	3125	39.7	
1	1977	25.1	2027	25.8	
≥2	3180	40.5	2711	34.5	

Abbreviation: COPD, chronic obstruction pulmonary disease; ENT, ear, nose, and throat; ER, emergency room; SD, standard deviation; USD, United States Dollar. ^†^ Standardized difference of ≥0.2 indicates a significant imbalance in a variable between exposure groups.

**Table 2:** Postoperative mortality and complications for dementia patients and controls.

Outcome of Interest	Dementia		Control		Perioperative Risk	*p*
	Event	Rate (%)	Event	Rate (%)	aOR (95% CI) ^†^	
30-day in-hospital mortality	52	0.7	30	0.4	1.71 (1.09–2.70)	0.0209
180-day in-hospital mortality	278	3.5	188	2.4	1.49 (1.23–1.81)	<0.0001
365-day in-hospital mortality	426	5.4	287	3.7	1.52 (1.30–1.78)	<0.0001
Major complications						
Pneumonia	170	2.2	115	1.5	1.48 (1.16–1.88)	0.0015
Urinary tract infection	242	3.1	158	2.0	1.59 (1.30–1.96)	<0.0001
Pyelonephritis	16	0.2	8	0.1	2.09 (0.89–4.91)	0.0892
Surgical site infection	38	0.5	30	0.4	1.27 (0.79–2.07)	0.3272
Sepsis	138	1.8	79	1.0	1.77 (1.34–2.34)	<0.0001
Acute myocardial infarction	15	0.2	12	0.2	1.27 (0.59–2.75)	0.5383
Stroke	64	0.8	61	0.8	1.07 (0.75–1.53)	0.6949
Pulmonary embolism	7	0.1	0	0.0	>999.99 (<0.01–>999.99)	0.8834
Deep vein thrombosis	4	0.1	5	0.1	0.86 (0.23–3.32)	0.8321
Cardiac dysrhythmias	58	0.7	46	0.6	1.34 (0.90–1.98)	0.1514
Acute renal failure	52	0.7	38	0.5	1.36 (0.89–2.08)	0.1535
Postoperative bleeding	6	0.1	7	0.1	0.80 (0.26–2.42)	0.6878
Blood transfusion	299	3.8	229	2.9	1.32 (1.11–1.58)	0.0022
Admission to ICU	185	2.4	134	1.7	1.40 (1.12–1.76)	0.0036

Abbreviation: aOR, adjusted odds ratio; CI, confidence interval; ICU, intensive care unit. ^†^ Adjusted for all covariates listed in Table 1.

**Table 3:** Sensitivity analyses for 30-day in-hospital mortality in patients with dementia compared with controls.

Statistical Model	30-Day in-Hospital Mortality			
	cOR (95% CI)	*p*	aOR (95% CI) ^†^	*p*
Primary model	1.74 (1.11–2.73)	0.0161	1.71 (1.09–2.70)	0.0209
Model 1 ^a^	1.72 (1.07–2.77)	0.0265	1.78 (1.10–2.90)	0.0200
Model 2 ^b^	1.93 (1.16–3.19)	0.0110	1.95 (1.17–3.27)	0.0108
Model 3 ^c^	1.94 (1.19–3.15)	0.0075	1.96 (1.20–3.21)	0.0074

Abbreviation: cOR, crude odds ratio; aOR, adjusted odds ratio; CI, confidence interval. ^a^ Model 1: excluding patients with dementia diagnosis <1 year before surgery. ^b^ Model 2: excluding patients undergoing cardiovascular surgery. ^c^ Model 3: excluding patients diagnosed with any type of cancer before surgery. ^†^ Adjusted for all covariates listed in Table 1.

**Table 4:** Subgroup analysis for postoperative mortality and complications associated with dementia.

Subgroup	Dementia/ Control	*n*	Perioperative Risk ^†^			
			Event	Rate (%)	aOR (95% CI) ^‡^	*p*
Age ≥ 65 years	Dementia	7064	872	12.3	1.26 (1.13–1.40)	<0.0001
	Control	7118	707	9.9	Reference	
Age < 65 years	Dementia	799	65	8.1	0.99 (0.67–1.46)	0.9594
	Control	745	65	8.7	Reference	
Male	Dementia	3741	479	12.8	1.14 (0.99–1.32)	0.0679
	Control	3691	420	11.4	Reference	
Female	Dementia	4122	458	11.1	1.35 (1.16–1.56)	<0.0001
	Control	4172	352	8.4	Reference	
Senile dementia	Dementia	5805	718	12.4	1.27 (1.14–1.42)	<0.0001
	Control	7863	772	9.8	Reference	
Vascular dementia	Dementia	886	90	10.2	0.96 (0.76–1.21)	0.7189
	Control	7863	772	9.8	Reference	
Alzheimer’s dementia	Dementia	416	53	12.7	1.45 (1.07–1.96)	0.0167
	Control	7863	772	9.8	Reference	
Dementia medication	Dementia	696	59	8.5	0.92 (0.70–1.22)	0.5769
	Control	7863	772	9.8	Reference	
No dementia medication	Dementia	7167	878	12.2	1.27 (1.14–1.41)	<0.0001
	Control	7863	772	9.8	Reference	
Dementia diagnosis ≥ 5 years	Dementia	1756	173	9.9	1.03 (0.86–1.23)	0.7390
	Control	7863	772	9.8	Reference	
Dementia diagnosis < 5 years	Dementia	6107	764	12.5	1.30 (1.17–1.45)	<0.0001
	Control	7863	772	9.8	Reference	
General anesthesia	Dementia	4289	482	11.2	1.28 (1.11–1.47)	0.0009
	Control	4298	384	8.9	Reference	
Regional anesthesia	Dementia	2320	267	11.5	1.32 (1.09–1.60)	0.0052
	Control	2359	215	9.1	Reference	

Abbreviation: aOR, adjusted odds ratio; CI, confidence interval. ^†^ Includes 30-day in-hospital mortality, pneumonia, urinary tract infection, pyelonephritis, surgical site infection, sepsis, acute myocardial infarction, stroke, pulmonary embolism, deep vein thrombosis, cardiac dysrhythmias, acute renal failure, postoperative bleeding, blood transfusion, and admission to ICU. ^‡^ Adjusted for all the covariates listed in Table 1.

## Supplementary Materials

The following are available online at https://www.mdpi.com/1660-4601/17/22/8431/s1, ICD-9-CM codes of comorbidity and outcome.
