# Supplementary material for: Preexisting Dementia Is Associated with Increased Risks of Mortality and Morbidity Following Major Surgery: A Nationwide Propensity Score Matching Study
Source: Int J Environ Res Public Health. 2020 Nov 14;17(22):8431. doi: 10.3390/ijerph17228431 (PMC7696268; doi:10.3390/ijerph17228431)
Supplement: Supplementary file 1 [file ijerph-17-08431-s001.pdf]

ICD-9-CM codes of comorbidity and outcome

Comorbidity	ICD-9-CM code
Hypertension	401-405
Diabetes mellitus	250
Ischemic heart disease	410-414
Atherosclerosis	440
Cardiac dysrhythmias	427
Heart failure	428
Liver cirrhosis	571.2, 571.5, 571.6
Chronic obstructive pulmonary diseases	490, 491, 496
Chronic kidney disease	585
Cerebrovascular disease	430-438
Parkinson's disease	332
Malignancies	140-208, 230-234
Sarcopenia	728.2
Obesity	278
**Outcome**	**ICD-9-CM code**
Pneumonia	480-486
Urinary tract infection	599.0
Pyelonephritis	590
Surgical site infection	682, 682.6, 682.9, 686.8, 686.9, 998.5, 998.51, 998.59
Sepsis	038
Acute myocardial infarction	410
Stroke	430-437
Pulmonary embolism	415.1
Deep vein thrombosis	451.11, 451.19, 451.2, 451.81, 451.9, 453.40-453.42, 453.8, 453.9
Cardiac dysrhythmias	427
Acute renal failure	584
Postoperative bleeding	998.1
